# Impact of Malt Bagasse Silage on Fungal Diversity, *Fusarium* Species, and Mycotoxin Contamination Under a Circular Economy Approach to Climate Change Mitigation

**DOI:** 10.3390/jof11070505

**Published:** 2025-07-04

**Authors:** Tania Valicenti, Carolina Manno, Juan Ignacio Poo, María Inés Dinolfo, Mauro Martínez, Andrea Enriquez

**Affiliations:** 1 Grupo de Suelos, Agua y Ambiente, Instituto de Investigaciones Forestales y Agropecuarias Bariloche (CONICET-INTA), San Carlos de Bariloche CP 8400, Río Negro, Argentina; taniavalicenti@gmail.com (T.V.); andreaenri@gmail.com (A.E.); 2 Instituto de Biología Funcional y Biotecnología (BIOLAB), INBIOTEC-CONICET, UNCPBA-CICPBA, Facultad de Agronomía, Universidad Nacional del Centro de la Provincia de Buenos Aires (UNCPBA), Azul CP 7300, Buenos Aires, Argentina; caromanno1997@gmail.com (C.M.); inesdinolfo@azul.faa.unicen.edu.ar (M.I.D.); 3 Laboratorio de Toxicología-Plataforma Analítica, Instituto de Innovación para la Producción Agropecuaria y el Desarrollo Sostenible INTA-CONICET, Balcarce CP 7620, Buenos Aires, Argentina; poo.juan@inta.gob.ar

**Keywords:** barley, climate change, *Fusarium*, malt bagasse, silo-bags, sustainability

## Abstract

Malt bagasse is the primary solid waste product from the brewing process, with notable environmental implications. Due to its nutritional value, it has potential as animal feed, primarily through ensilage. Alfalfa pellets can enhance this silage by adding digestible nitrogen and fibre. However, the high moisture content favours microbial contamination, particularly by fungi like *Fusarium*, which produces harmful mycotoxins. This study evaluated the impact of winter silage on fungal diversity, *Fusarium* presence, and mycotoxin contamination in malt bagasse, comparing the pre- and post-silage stages with the addition of alfalfa pellets. Results showed a diverse range of fungi, including *Mucor*, *Cladosporium*, *Fusarium*, and *Penicillium*, as well as yeasts. Fungal contamination was higher before silage, although the addition of alfalfa increased it after silage was produced. *Fusarium verticillioides* was the most common *Fusarium* species. Mycotoxin analysis detected DON (1.4 ppb) in only one sample. A two-month winter silage process under cold-temperate conditions appears to reduce fungal contamination and preserve feed quality. These findings support silage as a circular strategy to manage brewery waste safely, but further research and policy measures are needed to minimise biological risks in the brewing and livestock sectors amid climate change.

## 1. Introduction

In recent decades, overgrazing has accelerated the desertification process in arid and semiarid ecosystems, aggravated by the effects of climate change [1]. This fact has reduced the productivity of rangelands due to vegetation degradation, a decrease in forage species, shrub encroachment within the system, and an increase in bare soil [2]. Approximately 40.00% of the global population resides in these regions and relies on extensive livestock farming based on natural grasslands for economic sustenance and food sources [3,4]. However, the pressure on rangelands and climate change threaten the sustainability of this activity. In this context, strategic supplementation has emerged as a key tool to address critical periods, such as times of forage scarcity due to adverse climatic conditions (droughts or snowfalls) or higher nutritional demand from livestock, while preserving natural resources.

Another major challenge today is the efficient management of solid waste generated during food processing, which has significant environmental impacts, especially when its final destination is an untreated landfill. A notable case is malt bagasse, the primary waste product of the brewing industry, which is produced at both a homebrew scale, in microbreweries, and at an industrial level [5,6]. It is estimated that from 100 L of beer produced, 20 kg of dry malt bagasse is generated, representing about 85% of the total solid waste produced during the beer brewing process [6,7]. Global beer production is estimated at 1.89 million hectolitres annually; therefore, based on this approximation, the amount of discarded malt bagasse is not negligible worldwide, amounting to approximately 3.70 million tons per year [8,9]. Due to its high moisture and protein content, the improper management of malt bagasse can lead to significant environmental problems through greenhouse gas (GHG) emissions and/or nutrient release [6].

The circular economy proposes solutions to these environmental and resource issues by reintegrating waste or by-products into the production chain, such as alternative or unconventional forages referred to as “*Ecofeed*” [10]. This approach promotes the reduction in food waste, decreases greenhouse gas (GHG) emissions, and helps mitigate overgrazing, aligning with the sustainability goals of the 2030 Agenda. In this context, extended industrial waste storage could be a quick solution for agricultural regions where animal forage is scarce during unfavourable seasons, such as winter [11]. However, food safety and availability must be ensured for use as forage.

Malt bagasse has shown great potential as a dietary supplement due to its high nutritional value for animal feeding; however, its high moisture content makes its long-term storage difficult, and its low fibre content limits its use as an exclusive supplement. To address these challenges, ensiling in plastic silo-bags emerges as an economical and efficient solution, as it creates an airtight environment that preserves the quality of the stored material, minimising quantity and quality losses [12,13]. This method, widely adopted in global agricultural systems, offers logistical advantages and is a viable alternative to traditional storage methods [14]. Complementing malt bagasse with alfalfa (*Medicago sativa* L.) in pellet form is an ideal strategy to improve animal diets, as alfalfa provides fibre, high nitrogen digestibility, and practical advantages for distribution and management [15,16]. This animal feed could be a promising option for combining with malt bagasse and storing the mixture in plastic silo-bags for an extended period [17].

Despite the conservation advantages of plastic silo-bags, evidence suggests that several fungal species may grow in this semi-controlled environment, requiring a high CO_2_ concentration (>75%) to inhibit fungal growth and mycotoxin production [18]. Several fungal species have been isolated from stored cereal grains, with the most relevant genera being *Alternaria*, *Aspergillus*, *Cladosporium*, *Fusarium*, and *Penicillium*, among others [19,20,21]. Consequently, *Fusarium* species are among the most significant threats to the food chain and food safety. Food or feed safety refers to the conditions and practices that maintain the quality of food to prevent contamination and foodborne illnesses, ensuring that food is safe for human or animal consumption [22]. This concern is mainly due to the production and accumulation of mycotoxins such as deoxynivalenol (DON) and its acetylated derivatives (3-ADON and 15-ADON), fumonisins (FBs), and zearalenone (ZEA) [21,23,24]. However, the impact of fungal species and mycotoxin contamination on grain quality after the ensiling process remains unclear. Therefore, the present work aimed to evaluate the combination of malt bagasse and alfalfa pellets during storage in plastic silo-bags and assess fungal diversity, *Fusarium* species occurrence, and mycotoxin contamination to verify its food safety.

## 2. Materials and Methods

### 2.1. Silage Conditions

A winter storage trial was conducted between July and September 2023 at the “Dr. Grenville Morris” Experimental Agricultural Station of the National Institute of Agricultural Technology (INTA) in San Carlos de Bariloche, Argentina (41°7′22″ S; 71° 15′5″ W). The malt bagasse used in the trial was provided by a local brewery located in San Carlos Bariloche (Río Negro, Argentina). Malt bagasse was obtained immediately after brewing an ale beer style, with a predominant proportion of light malts, such as pilsner and pale malts. For the present work, two microscale silage simulation treatments were conducted using plastic silo-bags. Micro silo-bags (30.00 kg) were made using a plastic fraction of a commercial silo-bag, composed of three polyethene layers of high and low density (IpesaSilo^®^, Sao Paulo, Brazil). The white plastic side was placed on the outside layer to reflect solar radiation, while the black side was placed on the inner side to provide opacity [25].

Three treatments (30.00 kg per bag) were evaluated per triplicate (three repetitions per each silage treatment): pre-silage malt bagasse (MB-PRE), silage of single malt bagasse (MB-POST), and a mixture of malt bagasse and alfalfa pellets (MB + AA-POST), composed of 20.00 kg of malt bagasse and 10.00 kg of alfalfa pellets per bag. Plastic silo-bags were stored under natural conditions (rainfed) for 60 days (July–August). Silage samples were collected at the beginning and the end of the silage treatment. Moreover, after finishing the silage process, the samples were dried at 40.00 °C to preserve them. Climatic data were obtained monthly from an automatic meteorological station located at the experimental site, including minimum (Tmin), mean (Tmean), and maximum (Tmax) temperatures, as well as rainfall (Rf) during the study period [26]. To characterise the ENSO (*El Niño-Southern Oscillation*) phases, the Oceanic Niño Index (ONI) was also retrieved monthly for each growing season [27].

### 2.2. Morphological and Molecular Identification

Fungal diversity was evaluated for each treatment. A serial dilution assay was performed to count the number of Colony Forming Units (CFU) per volume (mL). For this purpose, 10 g of each treatment was finely ground, mixed with 90 mL of peptone water, and shaken at 145 rpm for 25 min. Then, the solution was filtered by gravity through a 1 mm filter, and after filtration, serial dilutions from 10^−1^ to 10^−5^ were prepared. For each dilution, three repetitions per treatment were placed in Petri dishes containing 2.00% potato dextrose agar (PDA) per triplicate (three repetitions). The PDA medium contained 0.25 g of chloramphenicol, whereas the incubation conditions were 25 °C ± 2 °C for five days, with a photoperiod of 12 h/12 (light/darkness). After this period, the total number of fungal colonies was registered and classified morphologically (Supplementary File S1). The colonies with similar characteristics to those of *Fusarium* spp. were selected and placed on Petri dishes containing carnation leaf agar (CLA) for morphological identification [28]. The CLA medium was prepared using sterile distilled water, 15 g of agar, and 50 g of dried carnation leaves (*Dianthus caryophyllus*) per litre of medium. Morphological identifications were based on the pigmentation of the colony, chlamydospore production, conidia shape and size, and the quantity of micro and macroconidia production.

The molecular identification of *Fusarium* spp. was performed using DNA from a single representative monosporic isolate for each *Fusarium* species [29]. The PCR reactions were carried out using 10–20 ng of DNA in a total volume of 25 μL containing 10× reaction buffer, 0.50 μM of each primer, 0.03 mM of each dNTP (Genbiotech S.R.L.), 2.50 mM MgCl_2_ and 0.20 μL of *Taq* DNA polymerase (Inbio-Highway, Tandil, Argentina). For amplifications, *Fusarium* species-specific primers were used for *F*. *chlamydosporum* (FChla-F- 5′ ATA CCT ATA CGT TGC CTC G 3′, FChla-R- 5′ GAA ATG ACG CTC GAA CAG 3′) and *F. verticillioides* (FVERT1- 5′ GTC AGA ATC CAT GCC AGA ACG 3′, FVERT2- 5′ CAC CCG CAG CAA TCC ATC AG 3′) [30,31]. DNA amplifications were performed in an XP Thermal Cycler (Bioer Technology Co., Hangzhou, China) with the conditions described for each primer set. The DNA amplifications were examined by electrophoresis in 1.50% (*w*/*v*) agarose gels and repeated twice. Each reaction was run with a known *Fusarium* species from the Institute BIOLAB collection (Buenos Aires, Argentina) as a positive control. A sample without DNA was used as a negative control, and a 100 bp molecular marker was used as a DNA ladder.

### 2.3. Mycotoxin Quantification

For mycotoxin analysis, each sample was finely ground (approximately 500 g), and 2.50 g was placed in 50 mL centrifuge tubes for each duplicate. After that, 10 mL of extraction solution (CH_3_CN/H_2_O/CH_3_COOH/HCOOH; 78:19:2:0.5) was added, and the sample was homogenised, sonicated for 5 min, and finally centrifuged for 5 min at 3500 rpm at room temperature (24 °C). From the supernatant, a volume of 100 µL was transferred to a 2.00 mL Eppendorf tube, and a 1:10 dilution with distilled water was performed. Before analysis, samples were filtered through a 0.22 µm nylon membrane filter and placed into glass vials. Recovery tests using standard solutions were conducted, yielding high recovery rates and indicating minimal retention of analytes by the filter. Fourteen mycotoxins were identified and quantified using ultra-high-performance liquid chromatography coupled with tandem mass spectrometry (UHPLC-MS/MS). Calibration and validation data, including the use of certified standards, five-point calibration curves with R^2^ ≥ 0.99, recovery, repeatability, and matrix effect, were generated and used to ensure analytical reliability. The following mycotoxins were quantified using external standards: aflatoxins (B1, B2, G1, and G2) (AFLA B1-B2-G1-G2), diacetocyscirpenol (DAS), deoxynivalenol (DON) and its acetylated derivatives (3-ADON and 15-ADON), fumonisins (FB1 and FB2), nivalenol (NIV), penicillic acid (PA), toxin T-2 (T-2), and zearalenone (ZEA). One additional sample containing a known mycotoxin concentration was included with each set of ten samples as a quality control measure for the extraction process. For each treatment, mycotoxins were quantified per triplicate. The detection limit (LOD) was 0.80, 0.20, 0.50, 1.10, 2.00, 0.30, 0.40, 6.00, 0.32, 0.60, 0.20, 0.20, and 1.20 μg kg^−1^ for PA, DON, 3-ADON, 15-ADON, NIV, DAS, T-2, ZEA, FB1, FB2, and (AFLA B1-B2-G1-G2). Whereas the quantification limit (LOQ) was 2.80, 0.40, 1.60, 3.60, 4.00, 0.80, 1.20, 1.20, 2.00, 0.40, 0.40, and 4.00 μg kg^−1^ for PA, DON, 3-ADON, 15-ADON, NIV, DAS, T-2, ZEA, FB1, FB2, and (AFLA B1-B2-G1-G2).

### 2.4. Statistical Analysis

A generalised linear model (GLM) was performed using RStudio software (v.4.2.1) and the MASS package [32,33]. The response variable was the count of colony-forming units per gram of sample (CFU g^−1^), and the data were initially fitted to a Poisson regression model. Subsequently, a generalised linear mixed model (GLMM) was fitted using the lme4 package with repetitions treated as random effects to determine which model provided the better fit (evaluated by AIC from the stats package) [34]. Overdispersion was assessed using the performance package, which necessitated adjusting the model to a negative binomial distribution [35]. The main factors in this model were the silage treatment (T) and the fungal genera (F). An analysis of variance (ANOVA) was conducted, followed by a post hoc Tukey test (α = 0.05) to compare the means among treatments. Additionally, Pearson’s correlation among fungal genera was explored, partitioning by silage treatment. Regarding mycotoxin contamination, a log + 1 transformation was carried out due to the non-normality of the data, and the results were expressed as the mean ± standard error of the mean (SEM).

## 3. Results

### 3.1. Climatic Conditions

**Figure 1:**
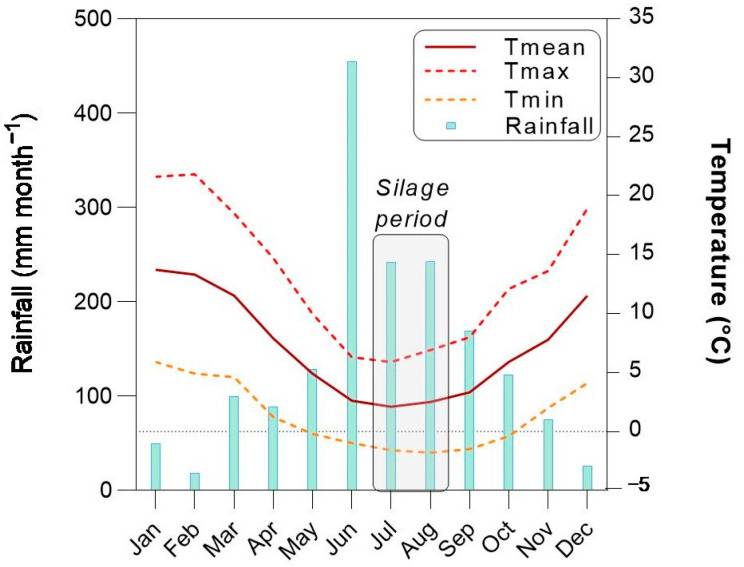
Climatic conditions for the experimental site. Rainfall (mm month^−1^, left axis) and air temperature (°C, right axis). Tmean: mean temperature. Tmax: maximum temperature. Tmin: minimum temperature.

According to the Oceanic Niño Index (ONI), the year 2023 was characterised as neutral at the beginning of the year, shifting to the “El Niño” phase since the April–May–June trimester (ONI values ranging from −0.70 °C to 2.00 °C). The total amount of rainfall was 1716 mm throughout the year, whereas during the silage process (July–August), only 485.30 mm was recorded (ca. 28.00%). Regarding mean temperature, the annual average was 7.25 °C, whereas, for the silage process, it was 2.30 °C. Annual minimum and maximum temperatures reached an average of 1.35 °C and 13.20 °C, respectively. During the silage period, the average minimum and maximum temperatures reached were −1.70 °C and 6.40 °C, respectively (Figure 1).

### 3.2. Fungal Diversity and Mycotoxin Contamination

Several fungal genera were isolated from each treatment at the different dilutions tested: *Cladosporium*, *Fusarium*, *Mucor*, *Penicillium*, *Rhizopus*, and yeasts (Supplementary File S1). Morphologically, *Cladosporium* spp. showed dark-pigmented, septate hyphae with branched conidiophores bearing chains of oval conidia, whereas *Mucor* spp. and *Rhizopus* spp. exhibited broad, aseptate hyphae and sporangia on erect sporangiophores, with rhizoids observed in *Rhizopus*. Regarding *Penicillium* spp., *these species* formed bluish-green colonies with branched conidiophores and chains of spherical conidia, whereas yeasts formed creamy colonies with unicellular, ovoid cells. As to *Fusarium* spp., these species exhibited hyaline, septate hyphae. *F. chlamydosporum* produced thick-walled chlamydospores, whereas *F. verticillioides* displayed slender monophialides and abundant microconidia in chains.

**Figure 2:**
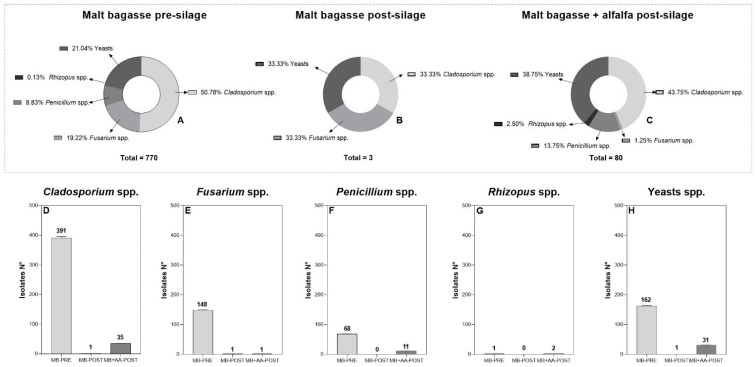
Fungal diversity along the silage process (**A**–**C**). Below: number of isolates for each fungal genus and partitioned by each stage (**D**–**H**).

The dilutions with more isolates reported were 10^−2^ (396 isolates) and 10^−3^ (300 isolates), followed by the original sample (98 isolates), 10^−1^ (33 isolates), 10^−4^ (17 isolates), and 10^−5^ (9 isolates) (Table 1). Significant differences were reported among the treatments, with a higher number of isolates observed in MB-PRE (770 isolates), followed by MB + AA-POST (80 isolates) and MB-POST (3 isolates) (Figure 2, Table 2). Moreover, significant differences were reported for fungal occurrence, whereas no interaction (T × F) was detected (Table 2). For the initial stage (MB-PRE), *Cladosporium* was the predominant genus isolated (c.a. 51.00%), followed by yeasts (c.a. 21.00%), *Fusarium* (c.a. 19.00%), *Penicillium* (c.a. 9.00%), and *Rhizopus* (c.a. 0.10%) (Figure 2A, Table 1). For the final stage (MB-POST), only one of the following genera was isolated: *Cladosporium*, *Fusarium*, and yeasts (33.33% each) (Figure 2B). Finally, for the mixture of malt bagasse and alfalfa pellets during the final stage (MB + AA-POST), the predominant genus was *Cladosporium* (c.a. 44.00%), followed by yeasts (c.a. 39.00%), *Penicillium* (c.a. 14.00%), *Rhizopus* (c.a. 3.00%), and *Fusarium* (c.a. 1.00%) (Figure 2C).

In general terms, *Cladosporium* spp. was predominant in all treatments evaluated, showing a higher number of isolates during the early stages (MB-PRE, 391 isolates), which decreased to 1 isolate and 35 along the silage process (MB-POST and MB + AA-POST, respectively) (Figure 2D). Yeasts were the second in relevance, reporting 162 CFU during the early stages (MB-PRE), whereas at late stages, only 1 and 31 isolates were reported (MB-POST and MB + AA-POST, respectively) (Figure 2H). *Fusarium* was the third relevant genus (see Section 3.3). Similarly, *Penicillium* showed a higher number of isolates during the early stages (MB-PRE, 68 isolates), whereas during the late stages, a reduction was reported (1 and 11 isolates, respectively) (Figure 2F). Finally, *Rhizopus* was the genus with the lowest occurrence, reporting only three isolates: one during MB-PRE and two during MB + AA-POST (Figure 2G, Table 2).

Regarding mycotoxin contamination, no contamination was registered during the silage process for 13 of the 14 mycotoxins evaluated. In only one sample (during the MB-POST period), DON was detected and quantified in one sample at a concentration of 1.40 ppb, which is above both the limit of detection (LOD = 0.20 ppb) and the limit of quantification (LOQ = 0.40 ppb). Therefore, the presence and concentration of this mycotoxin can be considered reliable within the analytical accuracy of the method employed (Supplementary File S2).

### 3.3. Fusarium Species and Fungal Correlation

**Figure 3:**
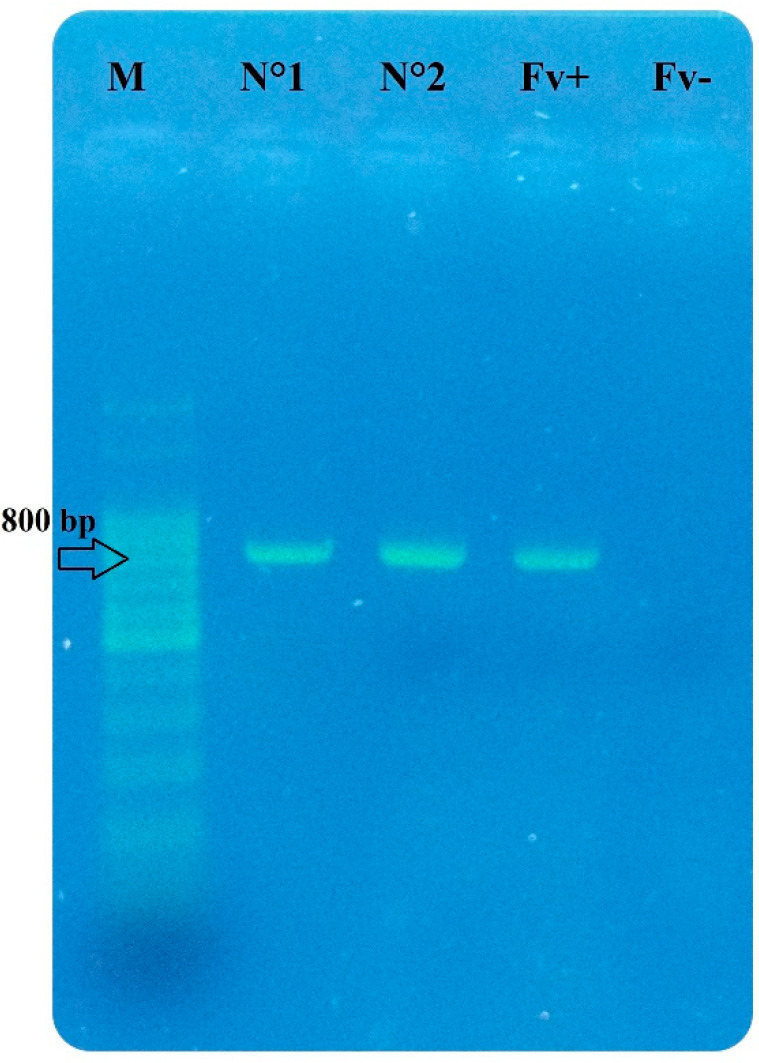
Electrophoresis agarose gel (1.5%) for molecular confirmation. M: molecular markers of 100 bp DNA ladder. N°1: isolate of *F. verticillioides* obtained from MB-PRE. N°2: isolate of *F. verticillioides* obtained from MB + AA-POST. Fv+: isolate of *F. verticillioides* of known sequence. Fv–: control. The arrow indicates the fragment amplified for *F. verticillioides* (800 bp).

**Figure 4:**
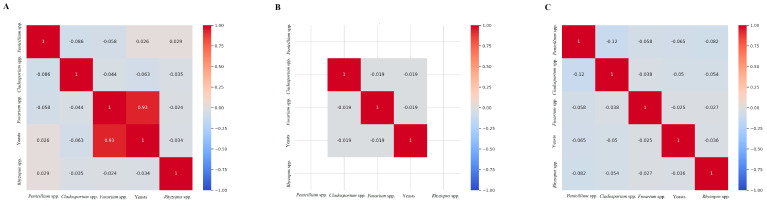
Pearson’s correlation for the different fungal genera isolated, partitioned by treatment: MB-PRE (**A**), MB-POST (**B**), and MB + AA-POST (**C**).

From the total number of isolates obtained (783), 150 (ca. 19.00%) correspond to the fungal genus *Fusarium*. During the early stages of the silage (MB-PRE), 148 isolates were obtained, whereas, for MB-POST and MB + AA-POST, only 1 isolate per stage was reported (Figure 2E). After morphological observation and molecular confirmation, two *Fusarium* species were identified: *F. verticillioides*, which was predominant (ca. 99.00%), and *F. chlamydosporum* (ca. 1.00%). More specifically, for MB-PRE, all the isolates (148) correspond to *F. verticillioides*, as well as the single isolate obtained from MB + AA-POST (Figure 3). For MB-POST, the only isolate obtained corresponded to *F. chlamydosporum*. Regarding fungal correlation, a significant positive correlation (R = 0.93) was observed between the occurrence of *Fusarium* and yeasts during the initial silage stage (MB-PRE). In contrast, no significant correlations among fungal genera were detected for the late silage stages (MB-POST and MB + AA-POST) (Figure 4).

## 4. Discussion

The present study aimed to evaluate the impact of the silage process on fungal diversity, the occurrence of *Fusarium* species, and mycotoxin contamination. More specifically, the effect of using plastic silo-bags was analysed after ensiling barley malt bagasse, either alone or in combination with alfalfa pellets, for two months. Climatic conditions recorded during the winter silage process indicated low temperatures and a moderate to high amount of precipitation. These climatic conditions were expected for the winter period in the experimental site (North Patagonia, Argentina) because the dominant climate in the study region is continental and cold-temperate, with a dry season usually during the summer, and precipitations ranging from 600 mm year^−1^ to 4000 mm year^−1^ influenced by the humid winds from the Pacific Ocean [36]. In this way, it is known that fungal occurrence, mycelial growth, and conidia germination are closely related to environmental conditions, mainly temperature, relative humidity, rainfall, and the availability of resources (e.g., nutrients) [37,38,39]. Thus, a low occurrence of fungal species and diversity was expected in our work due to the restricted conditions to which bagasse silage was subjected throughout the silage process.

In plastic silo-bags, grain temperature is a variable that primarily depends on environmental air temperature, the growing season (e.g., winter or summer), intra-day temperature fluctuations, and the agricultural region [25]. In general, cereal grains such as barley and wheat are ensiled immediately after harvest, typically during the summer season, when temperatures are high. Even under wet conditions, this process plays a crucial role in fungal development, jointly with temperature and water activity (a_w_) [24,25]. Conversely, in the present work, the silage process was carried out during the winter season (July and August), which contributed to low fungal development, low diversity, and, subsequently, scarce mycotoxin contamination. The higher isolate number reported during the early stages could be attributed to the malt origin, the climatic conditions during harvest and barley storage, and the controlled conditions to which the malt was subjected during the malting process [40]. In barley crops, microbial colonisation of the grains occurs in the field while the crop grows, with several bacterial and fungal genera usually present [41,42]. Similarly, in wheat-stored grains, the most relevant fungal genera include *Alternaria*, *Aspergillus*, *Cladosporium*, *Fusarium*, and *Penicillium* [20,21]. However, in barley, scarce information is available regarding the storage of malt or bagasse in plastic silo-bags and their potential impact on fungal diversity. Our results suggest a high occurrence of fungal genera, including *Mucor*, *Cladosporium*, yeasts, *Fusarium*, *Penicillium*, *and Rhizopus.* In agreement, Gonzalez Pereyra et al. (2011) isolated similar fungal genera in spent grains (without the silage process), with a high occurrence of *Mucor* spp. (100%), yeasts (100%), *Fusarium* spp. (30%), followed by *Aspergillus* spp. (27%), and in lesser proportion, *Alternaria* spp., *Cladosporium* spp., *Geotrichum* spp., and *Penicillium* spp., with some of these genera not being detected in our work [43]. Given the strong influence of the climate on fungal and microbial dynamics, further trials under different environmental conditions, such as the summer, are encouraged to evaluate potential shifts in community composition and associated mycotoxin risks.

In general, species from the *Mucor* genus are opportunistic saprophytes, usually isolated from several cereal grains and organic matter, with rapid development and colonisation. In contrast, *Cladosporium* spp. are cosmopolitan organisms, saprotrophs that affect cereals worldwide and cause black point disease [44,45]. Yeasts (wild yeasts, *Saccharomyces* spp. and non-*Saccharomyces* yeasts) are common microorganisms present in barley grains and husks but are not dangerous for animal and human diets [42,46]. As for *Fusarium*, species belonging to this genus are the most hazardous for animal and human consumption, producing several harmful mycotoxins as secondary metabolites [23]. These mycotoxins cannot be removed along the barley food chain during malting and the brewing process, remaining in higher concentrations in the wort and the final product (beer), as well as in rootlets and spent grains [47,48,49]. Concerning *Penicillium* and *Rhizopus*, their presence in this study was not relevant, particularly since no mycotoxins typically associated with *Penicillium*—such as citrinin, ochratoxin A (OTA), or penicillic acid—were detected [50]. Similar findings were reported for barley cultivated in Europe, where low levels of contamination with *Aspergillus* and *Penicillium* were observed, while genera such as *Cladosporium*, *Dreschlera*, *Epicoccum*, and *Fusarium* were consistently detected in all samples tested [51].

In our study, *F. verticillioides* was the most frequently detected *Fusarium* species, particularly during the pre-silage stage. This fact suggests that contamination likely occurred earlier in the production chain, either during the crop cycle, at harvest, throughout storage, or during the malting process. Although *F. graminearum* and *F. poae* are among the most commonly reported species in barley across South America, the detection of *F. verticillioides* is unsurprising, given its widespread presence under zero-tillage systems worldwide [52,53,54]. Furthermore, barley crops are often included in crop rotations with maize, a common host for several *Fusarium* species, such as *F. verticillioides*, *F. subglutinans*, and *F. proliferatum* [55]. Under temperate field conditions, fungal structures such as mycelium, spores, and resistant forms can survive between seasons in maize residues, soil, or alternative hosts, including weeds [56,57]. Several studies have reported that barley grains are contaminated with *F. verticillioides*, suggesting that this pathogen may be a common etiological agent in this crop, depending on the agricultural region and climatic conditions [58]. Supporting this, *F. verticillioides* was found to be more prevalent than *F. graminearum* in barley crops grown in Serbia, with only low levels of fumonisin contamination reported (average 2.40 µg/kg) [51].

Another main reason contributing to the higher occurrence of *F. verticillioides* in our study could be its adaptive advantages over other *Fusarium* species. For instance, *F. verticillioides* can grow under low water activity (a_w_ = 0.860) and lower minimum temperatures (4 °C), compared to *F. graminearum*, which gives it a competitive edge under less favourable environmental conditions [59,60]. Low water activity (a_w_ < 0.60) can severely limit the growth of bacteria, fungi, and yeast species in barley grains and malt, thereby restricting their proliferation and increasing the risk of mycotoxin contamination [61]. Thus, grain humidity becomes relevant from the field, where grain conserved with high moisture (<14%) can be affected by *Aspergillus*, *Fusarium*, and *Penicillium* species [62]. After the mashing process, malt bagasse has a high humidity content, which makes immediate silage or drying (at temperatures above 100 °C for up to 3 h after mashing) essential to reduce its humidity below 15% and ensure extended preservation periods [62].

Mycotoxins pose a significant global food safety concern due to their toxicological effects on human and animal health, their frequent co-occurrence in feed, and the impact of climate change on the emergence of new mycotoxins [63,64]. They also lead to economic losses by affecting the feed supply chain and livestock productivity [65]. Since the 1960s, around 100 countries have established regulatory limits for aflatoxins (AFs), deoxynivalenol (DON), fumonisin B1 (FB1), fumonisin B2 (FB2), ochratoxin A (OTA), T-2 toxin, HT-2 toxin, and zearalenone (ZEA). The European Community (2002) established strict limits for AFs (200 µg/kg), OTA (5 µg/kg), and ZEA (1000 µg/kg). In contrast, the FDA regulations are less stringent, with limits for AFs (300 ppb) and FBs (100 ppb) but no maximum levels for OTA and ZEA [66]. In MERCOSUR countries, only AFs in maize and peanuts (20 µg/kg) are regulated, leaving other mycotoxins unmonitored [63]. For animal feed, FBs are usually prevalent in maize grains and by-products, followed by DON, AFs, and ZEA. In wheat, DON, OTA, T-2, and ZEA are the most commonly found mycotoxins [63,64]. In barley, the EC and FDA commonly regulate ergot and derived alkaloids, establishing maximum levels of up to 150 µg/kg [66]. However, for other mycotoxins, scarce information is available in barley, leaving them unregulated and potentially underestimating their potential damages. North Patagonia, Argentina, is not the exception, with few works reporting mycotoxins in barley and malt bagasse destined for animal feed [62]. In this context, Asurmendi et al. (2013) reported that during the 2010/2011 growing season, 31% of the malt bagasse samples were contaminated with AFs (up to 50 ppb), with the highest values observed during the summer months [67]. Similarly, Gonzalez Pereyra et al. (2011) reported mycotoxin contamination with AFB1 (up to 44 µg/kg) and FBs (145 µg/kg) in malt bagasse samples, while ZEA and other AFs (AFB2, AFG1, AFG2) were not detected [43].

In our study, a comprehensive analysis of 14 different mycotoxins was conducted, yet only one (DON) was detected, and in a single sample. This exceptionally low level of mycotoxin contamination may be primarily attributed to the lower winter temperatures recorded during the evaluation period. Supporting evidence from ensiling studies in other crops suggests that controlled atmospheric conditions (e.g., specific CO_2_/O_2_ ratios) and even sub-zero temperatures can significantly inhibit fungal growth and subsequent mycotoxin production [68,69]. Furthermore, acidic environments may contribute to the degradation of certain mycotoxins, in addition to potential interactions or adsorption processes involving the silage matrix [70,71]. Together, these factors likely played a crucial role in minimising the presence of mycotoxins in the samples evaluated. However, analysing mycotoxin contamination alone offers only a partial view of fungal behaviour, underscoring the ecological complexity and food safety implications behind variable toxigenic responses in different matrices and strains. This interpretation aligns with the patterns observed in our work and previous evidence. For instance, mycotoxin production by *fungal* species can vary among different isolates and substrates, even in the absence of visible growth, whereas host identity and evolutionary history can influence toxigenic expression [72,73]. Additionally, geographic and temporal shifts in contamination are associated with changes in fungal communities, while climate factors further influence mycotoxin risk [74,75]. These aspects likely explain the low occurrence of mycotoxin contamination reported in our study, although fungal diversity and *Fusarium* species varied broadly.

Regarding the impact of *Fusarium* species on silage safety, the high occurrence of *F. verticillioides* in malt bagasse during the pre-silage stage could pose a potential risk for long-term storage. Although mycotoxins produced by *F. verticillioides*, such as FBs, were not detected in the present study, favourable micro-conditions within the silo-bag could promote fungal development, severely impacting animal feed safety. Factors like insect infestation (e.g., *Sitophilus granarius*) causing grain damage and temperature increases, or oxygen entry due to silo-bag damage disrupting anaerobic conditions, can promote this fungal growth [76]. Our findings suggest that controlled conditions can moderately limit fungal growth and mycotoxin contamination, preserving bagasse safety. Except for DON, no mycotoxins were detected before, after ensiling, or with the addition of alfalfa pellets. The presence of DON could indicate the involvement of *F. graminearum* at some point during the silage process, although it may not have been detected with the methods employed in this study. Additionally, it is important to consider that other *Fusarium* species, such as *F. culmorum* or members of the *F. sambucinum* species complex (FSAMSC), including *F. asiaticum* and *F. pseudograminearum*, may also be present in malt bagasse treatments and contribute to DON production [77]. Moreover, future research, which quantifies fungal DNA, could utilise this approach to bridge the gap between traditional and modern detection techniques.

This study found a significant decrease in the occurrence of fungal and yeast species after ensiling compared to the pre-ensiling stages. This effect can be attributed to the micro-conditions generated within the plastic silo bag (CO_2_ increase, O_2_ reduction, and pH reduction) and the low temperature during the silage storage period. In the interstitial atmosphere, it is known that during the silage process, the concentration of CO_2_ increases from approximately 1.00% to 30.00%, while the concentration of O_2_ decreases from approximately 18.00% to 2.00% [78]. However, evidence suggests that several fungal species can grow under normal silage conditions, requiring high CO_2_ concentrations (ca. 75.00%) for inhibition [18]. Adding alfalfa pellets to malt bagasse silage appeared to increase fungal contamination compared to pure malt silage; however, more studies are deemed necessary to confirm this trend. Alfalfa is known to be susceptible to infections caused by *Alternaria*, *Cladosporium*, *Fusarium*, *Phoma*, and yeasts during its growth cycle. However, the physical conditions in early silage (the first eight days) can severely restrict the growth of this pathogen [79].

## 5. Conclusions

This study offers an initial insight into how ensiling affects fungal diversity, *Fusarium* diversity, and mycotoxin contamination in malt bagasse in North Patagonia, Argentina. Our findings reveal a high occurrence of fungal genera, including *Mucor*, *Cladosporium*, *Fusarium*, and *Penicillium*, as well as yeasts. *Fusarium verticillioides* was the dominant *Fusarium* species despite low mycotoxin levels. In the regular cold climate of Northern Patagonia, the two-month ensilage period seems beneficial for ensuring animal feed safety and preserving bagasse quality. Adding alfalfa pellets may increase post-ensiling fungal contamination. However, further investigations are warranted for a better understanding of: (i) how changes in the dynamics of the fungal community depend on factors such as temperature, controlled atmosphere, silage acidification, and matrix adsorption; and (ii) how these factors subsequently influence fungal growth and mycotoxin contamination throughout the storage period. Future studies should also consider the impact of varying environmental conditions (e.g., temperate agricultural regions), interannual climatic variability (e.g., ENSO events), extended storage durations (>180 days), and increased sampling frequency to capture these changes more accurately. The impact of malt composition variations resulting from different beer recipes on fungal diversity and mycotoxin contamination also requires clarification, as these variations can directly influence fungal diversity and mycotoxin contamination. It is expected that including a proportion of maize, rice, rye, or wheat grains/malt could increase fungal contamination compared to the traditional recipe, which contains only malted barley. For the coming years, further research and policy development are crucial to address potential risks in the brewing and feed industries within the framework of a developing circular economy and a changing climate.

## Figures and Tables

**Table 1:** Isolate numbers partitioned by fungal genera for each treatment: malt bagasse during pre-silage (MB-PRE), malt bagasse after silage process (MB-POST), and the combination of malt bagasse and alfalfa pellets (MB + AA-POST).

Treatment	*Cladosporium* spp.						
	Without dilution	10^−1^	10^−2^	10^−3^	10^−4^	10^−5^	Total
MB-PRE	1	1	356	28	4	1	391
MB-POST	0	0	0	0	1	0	1
MB + AA-POST	0	0	28	3	3	1	35
	*Fusarium* spp.						
	Without dilution	10^−1^	10^−2^	10^−3^	10^−4^	10^−5^	Total
MB-PRE	0	1	8	130	7	2	148
MB-POST	0	0	0	0	0	1	1
MB + AA-POST	0	1	0	0	0	0	1
	*Penicillium* spp.						
	Without dilution	10^−1^	10^−2^	10^−3^	10^−4^	10^−5^	Total
MB-PRE	38	23	2	1	1	2	67
MB-POST	0	0	0	0	0	0	0
MB + AA-POST	5	3	1	1	1	0	11
	*Rhizopus* spp.						
	Without dilution	10^−1^	10^−2^	10^−3^	10^−4^	10^−5^	Total
MB-PRE	0	0	0	0	0	1	1
MB-POST	0	0	0	0	0	0	0
MB + AA-POST	0	0	1	0	0	1	2
	*Yeasts*						
	Without dilution	10^−1^	10^−2^	10^−3^	10^−4^	10^−5^	Total
MB-PRE	24	2	0	126	0	0	152
MB-POST	0	1	0	0	0	0	1
MB + AA-POST	30	1	0	0	0	0	31

**Table 2:** Analysis of variance for fungal occurrence (CFU) regarding the treatment and the fungal genus reported.

Source of Variation	LR Chisq	Df	Pr (>Chisq)
Treatment (T)	31.949	2	1.15 × 10^−7^ *
Fungal genus (F)	30.069	4	4.73 × 10^−6^ *
T × F	12.842	8	0.1174
Adjusted mean (log-transformed)			
Treatments		Fungal genus	
MB-PRE	0.1391 ^ab^	*Cladosporium* spp.	0.4440 ^a^
		Yeasts	0.3170 ^a^
MB-POST	>0.0001 ^b^	*Fusarium* spp.	0.0980 ^b^
		*Penicillium* spp.	0.0007 ^c^
MB + AA-POST	1.0678 ^a^	*Rhizopus* spp.	0.0001 ^c^

LR Chisq: likelihood ratio Chi-square test. Df: degree of freedom. Pr (>Chisq): Chi-square probability. * Significant differences (α = 0.05). Different letters indicates significant differences.

## Data Availability

The original contributions presented in this study are included in the article/Supplementary Materials. Further inquiries can be directed to the corresponding author.

## Supplementary Materials

The following supporting information can be downloaded at: https://www.mdpi.com/article/10.3390/jof11070505/s1, Supplementary File S1. Fungal diversity on APG (2%) for the different treatments, repetitions, and dilutions: MB-PRE (A, B, and C); MB-POST (D, E, and F), and MB+AA-POST (G, H, and I). For each picture, dilutions varied from left to right (without dilution, 10^−1^, 10^−2^, 10^−3^, 10^−4^, and 10^−5^). Supplementary File S2. Mycotoxin contamination for each sample evaluated (ppb), partitioned by repetition (1, 2, and 3), and their relative LOD and LOQ limits. Dash (-): not detected.

## Abbreviations

3-ADON	3-acetyl deoxynivalenol
15-ADON	15-acetyl deoxynivalenol
AF	aflatoxin
a_w_	water activity
CFU	colony forming units
CO_2_	carbon dioxide
FB1	fumonisin B1
FB2	fumonisin B2
FDA	Food and Drug Administration
DAS	diacetocyscirpenol
DON	deoxynivalenol
ENSO	El Niño Southern Oscillation
MB-PRE	malt bagasse pre-silage treatment
MB-POST	malt bagasse post-silage treatment
MB + AA-POST	malt bagasse and alfalfa pellets post-silage treatment
NIV	nivalenol
O_2_	oxygen
OTA	ochratoxin-A
PA	Penicillic acid
T-2	toxin T-2
ZEA	zearalenone
