# Supplementary material for: Impact of Malt Bagasse Silage on Fungal Diversity, Fusarium Species, and Mycotoxin Contamination Under a Circular Economy Approach to Climate Change Mitigation
Source: J Fungi (Basel). 2025 Jul 4;11(7):505. doi: 10.3390/jof11070505 (PMC12298717; doi:10.3390/jof11070505)
Supplement: Supplementary file 1 [file jof-11-00505-s001.zip › jof-3731573-supplementary.pdf]

Supplementary File S1. Fungal diversity on APG (2%) for the different treatments, repetitions, and dilutions: MB-PRE (A, B, and C); MB-POST (D, E, and F), and MB+AA-POST (G, H, and I). For each picture, dilutions varied from left to right (without dilution, 10^{-1}, 10^{-2}, 10^{-3}, 10^{-4}, and 10^{-5}).

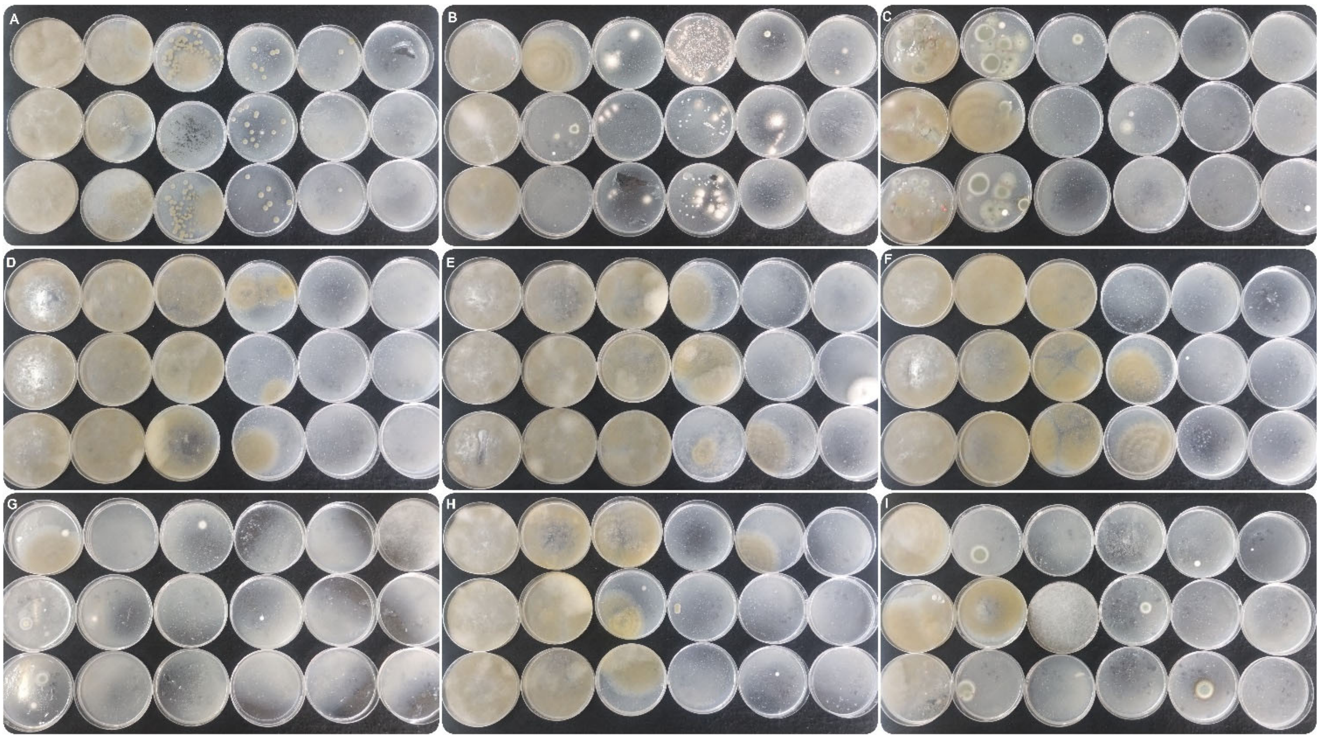

Supplementary File S2. Mycotoxin contamination for each sample evaluated (ppb), partitioned by repetition (1, 2, and 3), and their relative LOD and LOQ limits. Dash (-): not detected.

	LOD	LOQ	MB-PRE			MB-POST			MB+AA POST		
Mycotoxin	(ppb)	(ppb)	1	2	3	1	2	3	1	2	3
15-ADON	1.10	3.60	-	-	-	-	-	-	-	-	-
3-ADON	0.50	1.60	-	-	-	-	-	-	-	-	-
AFLA-B1	1.20	4.00	-	-	-	-	-	-	-	-	-
AFLA-B2	1.20	4.00	-	-	-	-	-	-	-	-	-
AFLA-G1	1.20	4.00	-	-	-	-	-	-	-	-	-
AFLA-G2	1.20	4.00	-	-	-	-	-	-	-	-	-
DAS	0.30	0.80	-	-	-	-	-	-	-	-	-
DON	0.20	0.40	-	-	-	-	1.40	-	-	-	-
FB1	0.20	2.00	-	-	-	-	-	-	-	-	-
FB2	0.20	0.40	-	-	-	-	-	-	-	-	-
NIV	2.00	4.00	-	-	-	-	-	-	-	-	-
PA	0.80	2.80	-	-	-	-	-	-	-	-	-
T-2	1.20	6.00	-	-	-	-	-	-	-	-	-
ZEA	0.32	1.20	-	-	-	-	-	-	-	-	-
